# Hypoplastic left heart syndrome

**DOI:** 10.1186/1750-1172-2-23

**Published:** 2007-05-11

**Authors:** Jean Anne Connor, Ravi Thiagarajan

**Affiliations:** 1 Cardiovascular Program, Children's Hospital Boston, USA; 2 Department of Cardiology, Division of Cardiovascular Critical Care, Children's Hospital Boston, USA

## Abstract

Hypoplastic left heart syndrome(HLHS) refers to the abnormal development of the left-sided cardiac structures, resulting in obstruction to blood flow from the left ventricular outflow tract. In addition, the syndrome includes underdevelopment of the left ventricle, aorta, and aortic arch, as well as mitral atresia or stenosis. HLHS has been reported to occur in approximately 0.016 to 0.036% of all live births. Newborn infants with the condition generally are born at full term and initially appear healthy. As the arterial duct closes, the systemic perfusion becomes decreased, resulting in hypoxemia, acidosis, and shock. Usually, no heart murmur, or a non-specific heart murmur, may be detected. The second heart sound is loud and single because of aortic atresia. Often the liver is enlarged secondary to congestive heart failure. The embryologic cause of the disease, as in the case of most congenital cardiac defects, is not fully known. The most useful diagnostic modality is the echocardiogram. The syndrome can be diagnosed by fetal echocardiography between 18 and 22 weeks of gestation. Differential diagnosis includes other left-sided obstructive lesions where the systemic circulation is dependent on ductal flow (critical aortic stenosis, coarctation of the aorta, interrupted aortic arch). Children with the syndrome require surgery as neonates, as they have duct-dependent systemic circulation. Currently, there are two major modalities, primary cardiac transplantation or a series of staged functionally univentricular palliations. The treatment chosen is dependent on the preference of the institution, its experience, and also preference. Although survival following initial surgical intervention has improved significantly over the last 20 years, significant mortality and morbidity are present for both surgical strategies. As a result pediatric cardiologists continue to be challenged by discussions with families regarding initial decision relative to treatment, and long-term prognosis as information on long-term survival and quality of life for those born with the syndrome is limited.

## Disease name and synonyms

The congenital heart lesion more commonly known today as hypoplastic left heart syndrome (HLHS) was initially termed hypoplasia of the aortic tract complex by Lev in 1952 [1]. This initial description resulted from examination of a series of specimens found to have isolated hypoplasia of the aorta, hypoplasia of the aorta and ventricular septal defect, and hypoplasia of the aorta with aortic stenosis or atresia, with and without mitral stenosis or atresia. The series of Lev was followed by the study of Noonan and Nadas, who in 1958 first used the term hypoplastic left heart syndrome collectively to describe their series of specimens with multiple malformations involving left-sided structures of the heart [2].

## Definition and diagnostic criteria

Hypoplastic left heart syndrome refers to the abnormal development of the left-sided cardiac structures, resulting in obstruction to blood flow from the left ventricular outflow tract. In addition, the syndrome includes underdevelopment of the left ventricle, aorta, and aortic arch, as well as mitral atresia or stenosis. The severity of outflow obstruction, the left heart structures involved, and the degree of left ventricular and aortic hypoplasia, may vary among patients, resulting in a spectrum of patients with varying levels of severity [3].

## Epidemiology

The syndrome has been reported to occur in approximately 0.016 to 0.036% of all live births [4-7]. It accounts for 1 to 3.8 % of all congenital cardiac malformations [8]. Up to seven-tenths of cases are reported to occur in males [4]. The recurrence risk in siblings is 0.5%, with other forms of congenitally malformed hearts seen in 13.5% [9].

## Clinical description

Newborn infants generally are born at full term, and initially appear healthy. With closure of the arterial duct, the systemic perfusion becomes decreased, resulting in hypoxemia, acidosis, and shock. Usually, no heart murmur, or a non-specific heart murmur, may be detected. The second heart sound is loud and single because of aortic atresia. Often the liver is enlarged secondary to congestive heart failure.

A small subset of patients have restriction of blood flow from the left to right atrium because of an inadequate or absent atrial communication. Such restriction to flow of blood from the left to right atrium can result in severe left atrial hypertension, and decreased blood flow into the right atrium and the dominant right ventricle, resulting in decreased flows into both the systemic and pulmonary circulations. These patients present with cardiogenic shock and profound cyanosis at birth, and are likely die in the absence of an intervention, such as catheter-based or surgical septostomy, performed soon after birth, to relieve obstruction or to create a means of communication in the atrial septum, allowing decompression of the left atrium and free flow of blood from the left atrium into the right atrium.

## Etiology

The embryologic cause, as in the case of most congenitally malformed hearts, is not fully known. Early epidemiologic studies report a multifactorial influence to be the cause of up to 90% of cardiac anomalies, with a recurrence rate in further offspring of 2% to 6% [10]. Associated risk factors include maternal, gestational, and familial conditions. Fetal exposure to teratogens may also be a risk factor. Likewise, fetal exposure to active maternal infections, such as rubella, herpesvirus, coxsackievirus B5, and cytomegalovirus, may be a risk.

Chromosomal aberrations account for about 6% of all congenitally malformed hearts. Many genetic and hereditary diseases are associated with such congenital malformations, although the causative mechanism is unknown. Prospective studies using chromosomal analysis have suggested that some malformations may be the result of a single gene defect [11].

In the case of hypoplasia of the left heart, the resulting multiple anomalies may result from either the multifactorial factors described above, or from a reduction of left ventricular inflow or outflow during fetal development.

## Diagnostic methods

The most useful diagnostic modality is the echocardiogram, which may help confirm the diagnosis, and as well as diagnose basic variability in anatomic structures within this anatomically heterogenous population. Information collected should include the size of the inter-atrial communication, function of the atrioventricular valve, and the size of the ascending aorta, as these are useful measurements for stratifying the options for treatment. Although the electrocardiogram is non-specific, many patients display right ventricular hypertrophy, and have paucity of left ventricular forces. Chest radiographs are not diagnostic, and cardiac catheterization is only needed if an intervention such as creation or enlargement of the inter-atrial communication is required [12].

## Differential diagnosis

The clinical presentation may resemble those of neonates with other left-sided obstructive lesions where the systemic circulation is dependent on ductal flow. These conditions may include critical aortic stenosis, coarctation of the aorta, interrupted aortic arch. As with hypoplasia of the left heart, their clinical presentation is cardiogenic shock following closure of the arterial duct. Other non-structural cardiac diseases, with clinical presentation in a shock-like state, such as neonatal myocarditis and neonatal sepsis, should also be included in the differential diagnosis. These conditions can be easily differentiated by echocardiography.

## Genetic counseling

Upon diagnosis, both genetic counseling and testing should be offered to both parents. Multiple genetic syndromes have been reported, including Turner's syndrome, Noonan's syndrome, Smith-Lemli-Opitz syndrome, Holt-Oram syndrome, and many others [13,14]. Overall, one-quarter of all patients have an associated genetic disorder or major extracardiac abnormality.

## Antenatal diagnosis

The syndrome can be diagnosed by fetal echocardiography between 18 and 22 weeks of gestation [14-16]. Further evaluation should include genetic testing, and the examination for other extracardiac malformations. Once the diagnosis is made, the perinatal team should monitor growth and development of the other fetal organs. Vaginal delivery is often recommended, as long as the fetus has no signs of cardiac failure. Although the syndrome has not been found to have deleterious effects on labor and delivery, most health care professionals advise that the birth of the infant should occur in a cardiac surgical center.

## Management including treatment

Afflicted children require surgery as neonates, as they have duct-dependent systemic circulation. Currently, there are two major treatment modalities. These are primary cardiac transplantation, or a series of staged functionally univentricular palliations [17]. The functionally univentricular palliation typically includes three operations. The first stage of palliation, or the Norwood operation, is performed at birth. The second stage is a bi-directional Glenn operation, usually undertaken at 6 to 8 months of age. The third, and final, stage is the Fontan operation, which can be performed between the ages of 18 months and 4 years. The treatment chosen is dependent on the preference of the institution, its experience, and also preference. Connor *et al*., recently evaluated outcomes for 251 children during 1997, with 17 managed by primary cardiac transplantation, and 234 by stages palliation, and showed that death occurred more frequently in those undergoing primary cardiac transplantation, with 42% dying, compared with those undergoing the Norwood operation, of whom 35% died [18]. The mortality rate for children managed through primary cardiac transplantation does not include children who died waiting prior to cardiac transplantation. The increased risk of death in children waiting for transplantation, and the scarcity of donor organs during the neonatal period, has made this modality less favorable, but is still offered in a few centers in the United States of America [19].

For patients undergoing functionally univentricular palliation, leading to creation of the Fontan circulation, the highest risk of mortality is following the initial operation, with up to three-tenths of patients dying in some reported series [20]. The Norwood operation consists of constructing a new aortic root and arch, disconnecting the pulmonary trunk from the pulmonary circulation, and incorporating it into the systemic outflow tract. A modified Blalock-Taussig shunt, of 3 to 4 millimeters in diameter, is constructed to supply blood to the lungs. As pulmonary vascular resistance is lower than systemic vascular resistance, blood flows from the aortic root to the pulmonary circulation during diastole. This results in decreased diastolic blood pressure in the aortic root, and thus decreased coronary arterial perfusion. As a result, these children may suffer from myocardial ischemia, causing cardiac failure and sudden death after discharge from hospital following successful initial surgical palliation. Since blood flows into the pulmonary circulation in diastole, the fraction of cardiac output distributed to the pulmonary circulation is higher than the systemic circulation, and the blood returning from the pulmonary circulation creates a volume load to the dominant right ventricle [21]. Recent modifications of the Norwood operation, involving replacement of the systemic-to-pulmonary arterial shunt with placement of a cyro-preserved non-valved conduit directly from the right ventricle to pulmonary arteries, have resulted in better maintenance of diastolic blood pressure, and in some centers have improved both hospital mortality and mortality after discharge for children undergoing the initial stage of palliation [22]. The advantage of placement of a conduit, however, has still to be proven in a randomized control trial.

The second stage of palliation is called the bi-directional Glenn operation. This consists of anastomosis of the superior caval vein to the right pulmonary artery, and takedown of the systemic-to-pulmonary arterial shunt or conduit [23]. Results following the venous shunt are very good, and the overall operative mortality is reported to be from 2 to 5.4%. The third stage of palliation is construction of the Fontan circulation, which consists of routing the inferior caval venous blood through a conduit placed in the lateral wall of the right atrium into the pulmonary arteries, or through an extracardiac conduit. A fenestration is usually created between the medial wall of the baffle and the systemic atrium to allow decompression of the atrial conduit pathway into the systemic atrium. The fenestration is usually closed in the catheterization laboratory up to 1 or 2 years after the Fontan operation. Nowadays, the operative mortality for conversion to the Fontan circulation is also less than 5%. Cardiac catheterization is usually undertaken prior to both the second and third stages of palliation to study anatomical and physiological details, and to perform corrective interventions. Long-term and functional outcome data following the functionally univentricular palliation is currently being evaluated.

## Unresolved questions

A number of unresolved questions continue to surround management and treatment [24]. Despite advances in surgical techniques and medical therapies, those with the syndrome continue to have the highest mortality of all congenital cardiac malformations for infants less than one year of age [25], rivaling in this regard those with pulmonary atresia and intact ventricular septum, and those with isomerism of the right atrial appendages, or asplenia syndrome. Although survival following initial surgical intervention has improved over the last 20 years, pediatric cardiologists continue to be challenged by discussions with families regarding initial decision relative to treatment, and long-term prognosis [26-28]. This is due in part to the limited information available in short-term and long-term functional and cognitive behavior in these children. The information that is available suggests that, regardless of surgical approach, staged surgical reconstruction or transplantation, long-term functional and cognitive behavior is strongly related to initial condition at diagnosis, and hence is variable [29-31]. Prenatal diagnosis, fetal intervention, and its impact on survival after the first stage of palliation, are areas of current study [32-34]. The limited information available has suggested that infants diagnosed during the prenatal period are more likely to survive the initial stage of palliation as compared to those that are not diagnosed during the prenatal period [35]. Small controlled clinical studies are investigating intervention in the fetal period to prevent the development of the syndrome through the use of balloon dilation and placement of stents in areas of restriction or hypoplasia. Short-term outcomes for this prenatal intervention are variable, and continue to be studied [36,37].

Another ongoing discussion, less in the United States of America than in Europe, involves the option of no intervention, known as compassionate care, and otherwise described as passive euthanasia. Until 1980, when the Norwood procedure was introduced, compassionate care was the only option available to infants born with the syndrome. Recent studies seem to suggest most diagnosed infants now initially undergo a surgical intervention, but it is not clear if the option of lack of intervention is discussed with families at the time of initial pre- or postnatal diagnosis [38-49].

## Psycho social concerns

Comprehensive counseling by the health care team is a critical component of care for the family of a child born with hypoplastic left heart syndrome. A review of the current outcomes of surgical intervention, as well as studies describing neurological and developmental outcomes, will guide families in their choice of treatment, or lack of treatment. For families who elect to have surgical intervention, care for their afflicted child is associated with numerous hospitalizations, services of a multidisciplinary team of specialists, and use of both advanced and innovative technology. The significant financial burden, or out of pocket expenses, that the family will incur should also be part of the preparation. Additional outside support can be provided by directing families to support groups available for families who have a child with congenital cardiac disease, and other online resources.

## Conclusion

As a result of advanced technology, refined surgical techniques, and catheter-based interventions, the mortality in the short term has improved dramatically for children born with hypoplasia of the left heart. The syndrome, nonetheless, it is still considered one of the most complex congenital cardiac malformations to manage. Many unresolved questions regarding treatment, and long-term functional and cognitive outcomes, remain for these children. Further information on these topics is required appropriately to guide discussions with families regarding initial options for treatment, long-term survival, and quality of life.
